# Are Varicose Veins a Cause of Post Partum Hœmorrhage?

**Published:** 1864-02

**Authors:** J. W. Brooks

**Affiliations:** Chicago


### CHICAGO
### MEDICAL JOURNAL.

VOL. XXI.] FEBRUARY, 1864. [No. 2.

### ORIGINAL COMMUNICATIONS.

### ARE VARICOSE VEINS A CAUSE OF POST PARTUM HŒMORRHAGE?

By J. W. BROOKS, M. D., Chicago.

If the following suggestions are novel, they may not be wanting in utility, should they lead to a more thorough investigation, of post partum hœmorrhage, as connected with varicose, of the uterus, uterine appendages and lower extremities. At this point we may very properly take a brief glance at the venous anatomy of the uterus, and then follow with cases illustrative.

The veins of the gravid uterus are numerous and much dilated and flattened like sinuses, the largest of them are termed sinuses. They consist of the usual lining membrane of the venous system surrounded by the muscular fibres of the uterine parenchyma which invest them as a second coat. They are arranged in several planes, and those of each plane have frequent anastamoses with each other, whilst those of one plane inosculate with those of another plane, forming a grand venous network.

There is this anatomical peculiarity in the venous system of the uterus: Many of the veins open upon the internal surface of the uterus by well defined orifices. They transfix this surface very obliquely with valves at their orifices, which are especially numerous and large upon that portion of the uterine surface to which the placenta is attached.

The only valves belonging to the uterine venous system so far as the writer is aware, are those at the orifices, and those at the points of inosculation, between the veins of the different planes. We believe with Prof. Owens, that "the foramen by which a venous tube communicates with another lying immediately beneath it, in the uterus, is not in the sides, but in the floor of the superficial vein, and working down in it from above." We see the canal of the vein below partially covered by a semi-lunar falciform projection, formed by the lining membrane of the two venous tubes as they meet at a very acute angle, the lower tube always opening very obliquely into the upper. That these valvular arrangements, by obstructing the retrograde movement of the venous blood, operate efficaciously in preventing uterine hœmorrhage, cannot be doubted, in the various exigencies of pregnancy and parturition. Let a varicose condition of these veins exist, and what will be the inevitable consequence? The answer will be found by ascertaining after a fatal case of post partum hœmorrhage attended with varicose veins, the condition of the veins and sinuses, together with the condition of the valvular orifices and the valves at the points of inosculation.

Especially will the case appear clear if the placenta has been attached, so as to involve part of the healthy and part of the varicose side.

From several cases that have occurred to the writer, we will present three as representative ones:

Case 1st—Was called in consultation with Dr. F., June 11th, 1851, to Mrs. F——n, in consequence of post partum hœmorrhage. Found her in articulo mortis on arrival, breathing but a few times, labor having been terminated about one hour previously, the child a male, healthy. The history of the case as derived, was briefly this: The lady was married at twenty, and was at the time thirty-eight years of age. She had borne eight children previously, and enjoyed uniformly good health. Her labors had been comparatively easy and she had hitherto made good recoveries. The present labor had lasted about three hours, without unusual occurrence, till after the delivery of the placenta, when the blood flowed profusely and persistently; cold applied externally not checking it, and the uterus meantime alternately contracting and relaxing. Sectio. Cadaver, thirty-six hours after death. Body was well formed and developed, ex-sanguine. There were large varices extending from the foot to the inguinal region of the right side. Rigor Mortis was complete. On opening the abdomen the parts were normal, if we except the ex-sanguiousness, and the uterus: This was not fully contracted, and a marked difference existed in the two lateral halves. On the external part of the right side, embracing the right appendices uteri, the veins appeared much as they did in the labia externa and through the right lower extremity. Dividing the uterus on the mesian line, we found the placenta had been attached about equally on either side of the mesian line. The veins of the left side seemed in a healthy condition, the valves normal, and small coagula were found within the valves where the placenta had been attached. Not so the right side: Here the veins and sinuses appeared from twice to four times the size of the corresponding ones of the opposite side. The valves where the placenta had been attached were almost entirely effaced. There was scarcely a coagulum within the veins or sinuses. The valves at the points of inosculation between the planes were similarly diseased, while the veins appeared like the other varicose veins. The heart and other organs were perfectly healthy.

Case 2nd—Was called to Mrs. C———, March 10th, 1855. Found Dr. O——r in attendance. The child, a fine healthy girl, had been delivered about one hour and ten minutes. Dr.

O. reports flooding as having commenced soon after the birth of the child. It continuing, he depressed the head, elevated the pelvis, and applied cold to the uterine region without apparent good result. Alarming prostration supervened, upon which he introduced a piece of ice, enclosed in an oiled napkin with no relief. On my arrival death was evidently near. The veins of right leg, the mesh below Poupart's ligament and those of the right labia were almost universally in a varicose state. We attempted the use of solution of Sulphate Ferri as a styptic locally, but with very little result. Life was ebbing rapidly, and in just twenty minutes from my reaching the bedside she ceased to breathe.

The following brief history of the case was gleaned: She was married at 24, and was at the time 35 years of age. She had never seen a sick day except very slight indisposition attending five previous confinements with living healthy children. The first notice of varicose veins was during her fourth pregnancy, but there was no hœmorrhage during or after labor more than ordinarily attends. During the fifth the varices were much larger, but the labor was easy and the recovery good. During the sixth the varices were still increased, especially near the inguinal region, and she complained of a constant uneasiness in the right abdominal region, and her expression was that she had the same feeling in it that she had in the varicose veins below. In all other particulars she felt well.

Sectio. Cadaver, 30 hours post abeit. A beautifully developed female form presented, ex-sanguious. Traces of the varices were still to be seen on the limbs, &c. On opening the abdomen the appearances were nearly a fac simile of Case 1st. On section of the uterus we found the placenta had been three-fourths of it attached on the right side. To describe the appearances of the valves, sinuses, &c., would be to repeat what has already been described in the preceding case. Other than this every thing appeared perfectly healthy.

Case 3rd—Mrs. E——— was at the period spoken of 41

years of age and had been always delicate, and is of a nervo-sanguine temperament. She had borne nine children. During her eighth pregnancy some varices appeared on the right leg, and extended to a point just below Poupart's ligament, and involved the veins of the right labia. Her labor was natural, no unusual hœmorrhage attended and she made a good recovery. So also was the case in the ninth period of gestation and accouchment. She conceived again in May, 1858. In August was called to see her in consequence of her experiencing dull pain, deeply seated in the right inguinal region, and extending from thence downward, through the right side of the vagina, the right labia, and thence to the ancle. She says the pain is greatly relieved by the recumbent posture. On examination small varices were found just above the right malleolus externus, at and just below the poplitcal region, on the outside of the thigh at the junction of the lower and middle third of the femur, in the fold below Poupart's ligament, between the right external labia and the thigh, in the right labia, and in the venous system of the right side of the vagina, with a knotty feel of the right side ot the neck of the uterus. She was enjoying her ordinary health. Directed a flannel bandage one and a half inches wide to be applied to the limb before rising in the morning, to exercise moderately, and to recline a part of the time during the day. In November was called again. She then stated that she had found much relief by the course prescribed. She was at this time suffering from a sensation of weight and tenderness in the right abdominal region, and spoke very positively of her belief of its location in the womb, and that her child was on the other side.

Stethescopic and tactile examination soon convinced me that the placenta was attached on the right lateral half of the uterus. The sounds observed were peculiar, and not easily described. Fearing diseased valves and varicose veins, I directed her exercise to be gentle, her diet mild, with instructions to recline often, with the request to be summoned to her side on the first intimation of labor. If labor occurred with hœmorrhage, that her hips be raised and her head depressed. Labor commenced in accordance with her reckoning, Feb. 10th, 1859. Every thing progressed favorably until after the birth of a son, when moderate hœmorrhage commenced. The placenta remaining and little or no contraction of the uterus having taken place, we dropped the head and elevated the pelvis and extremities, and then tried to induce contraction by the application of ice to the hypogastrium, and at intervals grasping the uterus through the abdominal parieties. These means partially abated the hœmorrhage. In thirty minutes from the delivery of the child syncope approached and alarming prostration. I directed an assistant to compress the abdominal aorta, and proceeded to deliver the placenta, which was found abnormally attached to the right side of the uterus. On separating the attachment the hœmorrhage was fearful. The orifices of the bleeding vessels were manifest to the feel. With the withdrawal of the placenta the uterus contracted briskly and then relaxed, continuing alternately to contract and relax until in the course of twenty minutes it assumed near a normal size, with little wasting. Pressure was continued fifteen minutes longer, and then was very gradually removed.

She was retained in the above position for twenty hours, at the expiration of which time, the head and pelvis were brought to the same level. She made from this time a gradual, but finally a good recovery, and at this time, January, 1864, enjoys comfortable health. She has always a painful sensation of weight and fulness on the right portion of the uterus, &c., during and for one or two days previous to the catamenial flow. This discharge still being regular at the month.

